# Improving prediction accuracy in chimeric proteins with windowed multiple sequence alignment

**DOI:** 10.1016/j.csbj.2025.07.039

**Published:** 2025-07-23

**Authors:** Sanketh Vedula, Alex M. Bronstein, Ailie Marx

**Affiliations:** a Technion – Israel Institute of Technology, Haifa 32000, Israel; b Institute of Science and Technology Austria, Klosterneuberg 3400, Austria; c MIGAL – Galilee Research Institute, Kiryat Shmona 1101600, Israel; d Tel Hai Academic College, Upper Galilee 1220800, Israel

**Keywords:** Protein structure prediction, AlphaFold, Multiple-sequence alignment

## Abstract

A key step in protein structure prediction involves the detection of co-evolving pairs of residues, a signal for spatial proximity. This information is gleaned from multiple sequence alignment and underscores Alphafold’s structure prediction for almost every known protein. A simple means to create proteins beyond those found in nature, is by unnaturally fusing together two known proteins or protein parts. Here we demonstrate that structured peptides are predicted with significantly reduced accuracy when added to the terminal ends of scaffold proteins. Appending the multiple sequence alignment for the individual peptide tags to that of the scaffold protein often restores prediction accuracy. This work suggests that this windowed multiple sequence alignment approach can be a useful tool for predicting the structure of fused, chimeric proteins.

## Graphical Abstract

**Figure:**
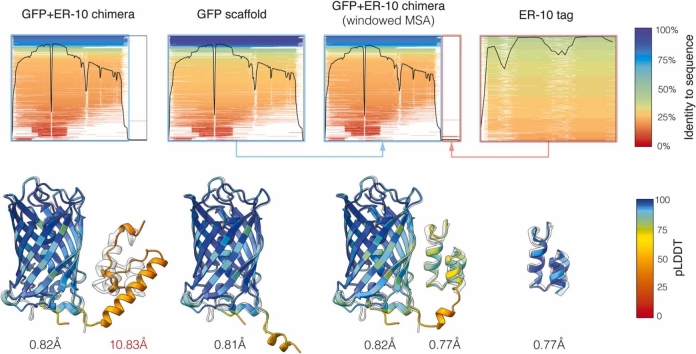

## 1 Introduction

Proteins have evolved over billions of years, accumulating sequence variations over time. Recognizing that coevolution of positions distant in the protein sequence is indicative of proximity in the protein structure revolutionized attempts to predict structure from sequence [12], [3], [5], [9]. This fundamental discovery has remained a cornerstone of state of the art, deep learning, prediction methods where the same key information is extracted as subtle signals from alignments of large numbers of sequences, and it is referred to as the multiple sequence alignment (MSA) step [6].

The critical importance of the MSA step in structure prediction raises questions about the reliability of predictions for non-natural proteins. In this work we consider chimeric proteins and show that contemporary protein structure prediction methods including AlphaFold-2 [6] and 3 [1] and ESMFold (Hayes et al., 2024) consistently mispredict the experimentally determined structure of small, folded peptide targets when presented as N or C terminus sequence fusions with common scaffold proteins. We find that for peptide targets and scaffold proteins predicted with high accuracy when presented as individual sequences, the accuracy of prediction for the target peptide deteriorates when presented as a fusion sequence with the scaffold protein. These pervasive errors point to a broader limitation in the ability of current models to inductively generalize beyond their training sets.

Investigating the origins of these inaccuracies, we identify the construction of the multiple sequence alignment (MSA) as the primary source of error. Specifically, the MSA based structural signals for the target protein are lost in the fused sequence form when using default MSA parameters. To address this shortcoming, we develop a Windowed MSA approach, which entails independently computing MSAs for the target and the scaffold that are then merged into a single alignment for structure prediction. This strategy avoids the artifacts introduced by attempting to align the entire chimeric sequence at once, while still retaining the essential evolutionary information for both the target peptide and scaffold.

Empirical validation of the windowed MSA procedure, conducted using AlphaFold-3 on a set of 408 fusion constructs, demonstrates a marked improvement in predictive accuracy. Specifically, windowed MSA produces strictly lower RMSD values than standard MSA in 65 % of these cases, without compromising the structural integrity of the scaffold. In the remaining cases, any increase in RMSD values is marginal and does not result in a visibly worse structural model, underscoring the robustness of the windowed MSA approach for chimeric protein modeling.

## 2 Methods

### 2.1 Dataset creation

To remove redundancy among the peptide sequences from McDonald et al. [10], we clustered them using a 50 % sequence similarity threshold and an 80 % bidirectional coverage threshold, where coverage is defined as the minimum of the query and target lengths. This process reduced the original set of 593 sequences (reported in McDonald et al. [10]) to 394 non-redundant sequences. Out of this non redundant set we selected only peptides predicted with high accuracy, namely an overall RMSD of (<1 Å) between the prediction and the experimentally determined structure. Peptide sequence having less than 2 MSA hits were removed. This process resulted in 51 peptide targets for in silico fusion to scaffold proteins. All combinations of the 4 scaffolds with 51 target peptides, attached once at N and C terminus, were generated resulting in total 408 unique sequences. We note that the scaffold sequences used correspond to that found in the crystal structures, 2IYD:B, 1PKW, 2B3P and 1MPB respectively, meaning that the SUMO and MBP proteins were truncated at the N terminus with respect to the native protein. Chimeric proteins were created by the addition of peptide tag sequences to the C and N terminus, individually, of the scaffold proteins. A small and flexible GLY-SER linker was inserted between the protein parts to alleviate any potential steric constraints in the concatenated sequences.

### 2.2 Structure prediction

We obtain AlphaFold-2 predictions by running ColabFold Mirdita et al. [11] and AlphaFold-3 predictions by running the source code locally (recently released by Abramson et al. [1]), using the same MSAs provided for AlphaFold-2 to ensure a like-for-like comparison. For ESMFold3 predictions, we use the recently-released ESM3 language model Hayes et al. [4], and its structure prediction head. For ESMFold3, we considered both iterative and argmax decoding as recommended in Hayes et al. [4]; we set the iterative decoding version as the main baseline because of its better accuracy. Accuracy of prediction was measured by calculating the RMSD between the experimentally determined peptide structure (specifically, the first structure in the NMR ensemble) and the peptide sequence region of the fusion protein.

### 2.3 Windowed MSA

For each the scaffold and tag regions, we generated MSAs using the MMseqs2 server via the ColabFold API (api.colabfold.com), searching against UniRef30 (release 2302; PDB100 230517). The scaffold sub-alignment included homologs spanning the scaffold sequence and explicitly incorporated the “GLY-SER” linker, while the peptide sub-alignment was built exclusively from peptide homologs. These sub-alignments were merged by concatenating scaffold and peptide MSAs with gap characters (-) inserted to fill non-homologous positions: peptide-derived sequences carry gaps across the scaffold region, and scaffold-derived sequences carry gaps across the peptide region, thus preserving the original alignment lengths and preventing spurious residue pairing. These finalized windowed MSAs were used as inputs to AlphaFold-2 and AlphaFold-3.

### 2.4 Molecular dynamics simulations

The PDB2PQR server [7] that was used to add hydrogen atoms and prepare the files for input to GROMACS, version 2020.2 [8]. The Amber 99sb-ildnp force field [2] was applied to normal amino acids and ions, and the SPC model was applied to water molecules. After solvation in a cubic box, the addition of Cl– and Na+ ions to balance the charge, energy minimization and heating to 300 K, the system was equilibrated under NVT and NPT conditions, each for 50 ps. Production runs of 50 ns were performed under NPT conditions, with a time step of 2fs. The temperature and pressure were maintained at 300 K and 1 bar.

## 3 Results

We created a large set of in silico fused proteins by adding the sequences of short, structured peptide targets at the N and C terminus of the following scaffold proteins, SUMO2, GST, GFP and MBP. The peptide targets were selected from a recent benchmark assessing the performance of AlphaFold-2 on peptide structure prediction McDonald et al. [10]. These peptides all have NMR determined structures, an advantage for assessing Alphafold performance since these models were not trained on NMR structures, preventing bias.

**Fig. 1:**
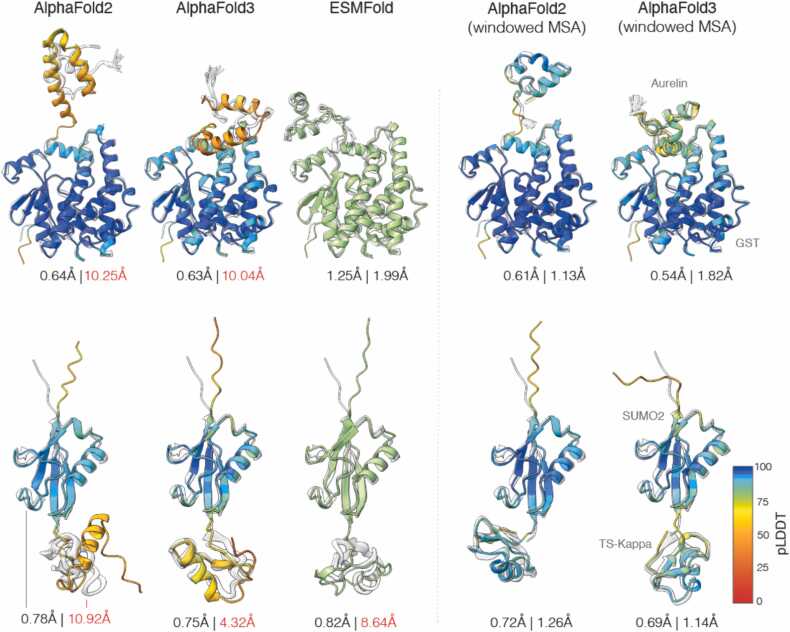
**The windowed MSA method restores the prediction accuracy of target proteins successfully predicted as an individual sequences and mispredicted as part of scaffold fused sequences using default MSA parameters.** Shown left-to-right are the predicted structures of two fusion sequences (first row: GST with 2LG4 fused to the C terminus; second row: SUMO with 1TSK fused to the N terminus+). AlphaFold-predicted structures are colored by the pLDDT confidence and ESMFold predictions are shown in green. Individual experimentally determined structures the scaffold and target peptides are superimposed in grey. Prediction accuracy is reported in terms of RMSD separately for the scaffold (left) and the target protein (right). While the scaffolds are invariably predicted accurately, target structures are mispredicted by the standard MSA-based AlphaFold (RMSD highlighted in red) and predicted very accurately with the proposed solution.

**Fig. 2:**
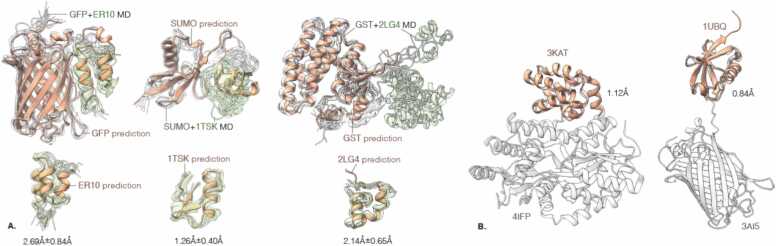
**Scaffold-peptide fusions are not expected to alter the structure of the isolated peptide**. (A) Molecular dynamics simulations show that although the peptide tag can move independent of the scaffold via the GS linker, the tag maintains the same structure as in the isolated state. Predicted structures are shown in pink (scaffold) and orange (tag) and structures from across the MD trajectory are shown in gray (scaffold) and green (tag). On the bottom row, alignment between the prediction and the MD derived structures is shown for the tag region of the fusion only. (B) Alignment between the experimentally determined fusion constructs and the experimentally determined structure of one of the protein components shows no alteration to the structure or interaction between the fused components. Shown on the left is the isolated structure of human NLRP1 CARD (PDB code:3KAT, shown in pink) aligned to the human NLRP1 CARD – MBP fusion structure (PDB code: 4IFP, shown in gray) and shown on the right is a isolated structure of ubiquitin (PDB code: 1UBQ, shown in pink) aligned to the ubiquitin – GFP fusion (PDB code: 3AI5, shown in gray). The RMSD values of the alignment are shown.

The fusion of structured proteins, scaffolds, to target proteins is common in experimental biology, enabling applications ranging from visualization (e.g., GFP) and solubility enhancement (e.g., SUMO), to affinity purification (e.g., GST, MBP). Target proteins appended to the N- or C-termini of the scaffold protein typically fold independently, and with minimal structural perturbation to either the scaffold or the target proteins. Vymětal et al. [15] recently curated a set of fused proteins having experimentally obtained high resolution X-ray structures for both the individual protein components and the fusion construct. In all cases, including MBP and GFP fusions, the individual components had a high structural similarity to the respective domains in the fusion construct (Fig. 2). To further justify our assumption that the free and fused conformations of the target peptides should be similar we ran molecular dynamics simulations of the chimeras shown in Fig. 1 finding that indeed the overall conformation of the targets do not change over the course of the simulation (Fig. 2).

### 3.1 Alphafold3 shows the highest accuracy in predicting the structure of peptide targets

**Fig. 3:**
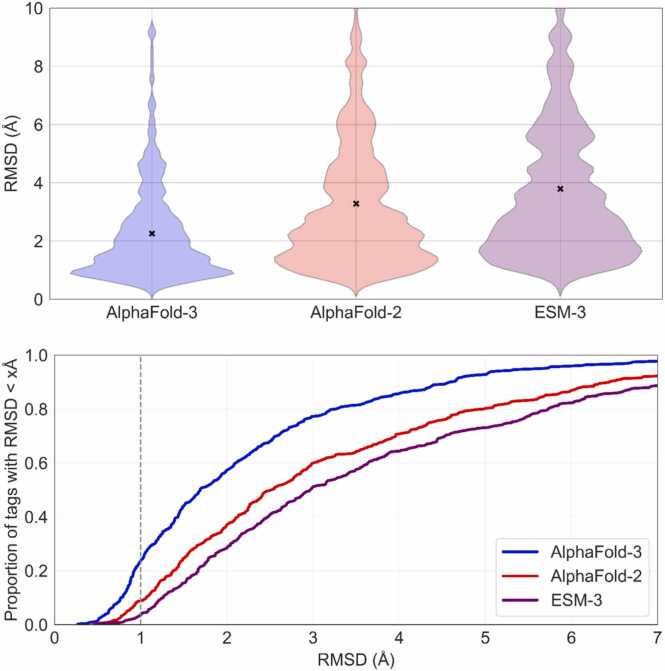
**Comparison of the accuracy of AlphaFold-3, AlphaFold-2 and ESMFold3 predictions on the peptide structure prediction benchmark**. AlphaFold-3 shows systematically higher accuracy compared to AlphaFold2 and ESMFold3; it achieves RMSD accuracy of < 1 Å for 90 out of 394 targets, when compared to AlphaFold-2 and ESMFold which produced 34 and 18 peptides that have lower than 1 Å RMSD, respectively.

Fig. 3 compares the performance of AlphaFold-2, AlphaFold-3, and ESMFold3 on the peptide structure prediction benchmark introduced in McDonald et al. [10]. Notably, AlphaFold-3 yields substantially more accurate predictions than both AlphaFold-2 and ESMFold, achieving an RMSD of less than 1 Å for 90 of the 394 targets. In comparison, AlphaFold-2 attains RMSD below 1 Å on only 34 targets, while ESMFold-argmax and ESMFolditerative reach this level of accuracy for just 18 and 21 targets, respectively. As ESMFold-iterative resulted in better performance, we choose this as the main ESMFold baseline.

### 3.2 All structure predictors lose accuracy in peptide prediction when this sequence is augmented to that of a scaffold protein

**Fig. 4:**
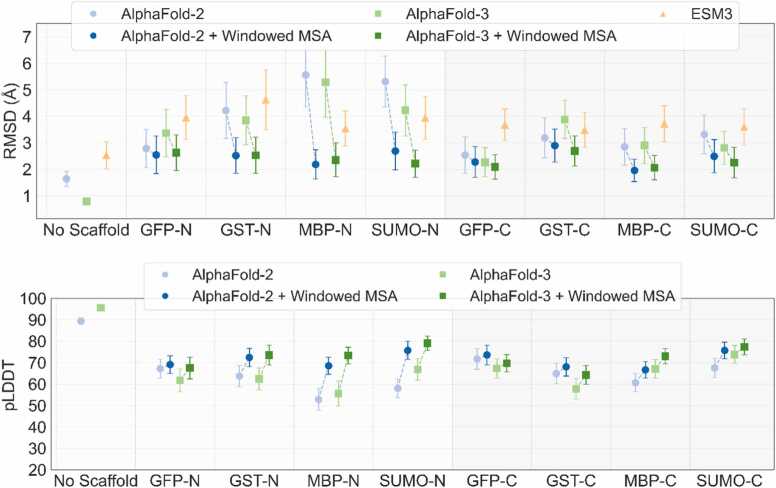
**Scaffold context reduces target peptide prediction accuracy.** The windowed MSA approach mitigates this effect and improves model confidence. Comparison of target peptide prediction accuracy (RMSD, top) and model confidence (pLDDT, bottom) across scaffold contexts. Results are presented as CI plots for both without scaffold (isolated targets, control) and with scaffold (targets fused to scaffold termini). Tags prediction accuracy drops sharply (RMSD increases) and pLDDT scores decline, indicating destabilized model confidence in the presence of a scaffold. The windowed MSA approach restores accuracy near control levels and elevates pLDDT scores, demonstrating improved structural modeling and confidence.

Considering only target peptides well predicted (RMSD <1 Å) by AlphaFold3, we measure how the accuracy of prediction changes in the context of a scaffold by augmenting the target peptide sequence to the scaffold termini. Representative results are presented in Fig. 1, and they demonstrate that AlphaFold-3, AlphaFold-2, and ESMFold predictions are worsened in the context of a scaffold protein, as observed by the largely increased RMSD between prediction and experiment at the peptide sequence region Fig. 4 presents a target-level breakdown of the ratio of the RMSD of the targets when predicted in scaffold context with respect to the RMSD when predicted in isolation. We notice that prediction accuracy of the peptide targets is worse when attached to the N terminus as compared to C terminus attachment. Using the windowed MSA approach the prediction accuracy of peptide targets is comparable for N and C termini attachment. Testing on a small number of peptide tag scaffold fusions we find that linker length does not affect prediction accuracy of the tag (Sup. Fig. 1) and neither does the addition of peptide tags to both termini of the scaffold (Sup. Fig. 2)

### 3.3 Appending the MSA of the peptide target to the scaffold restores prediction accuracy

**Fig. 5:**
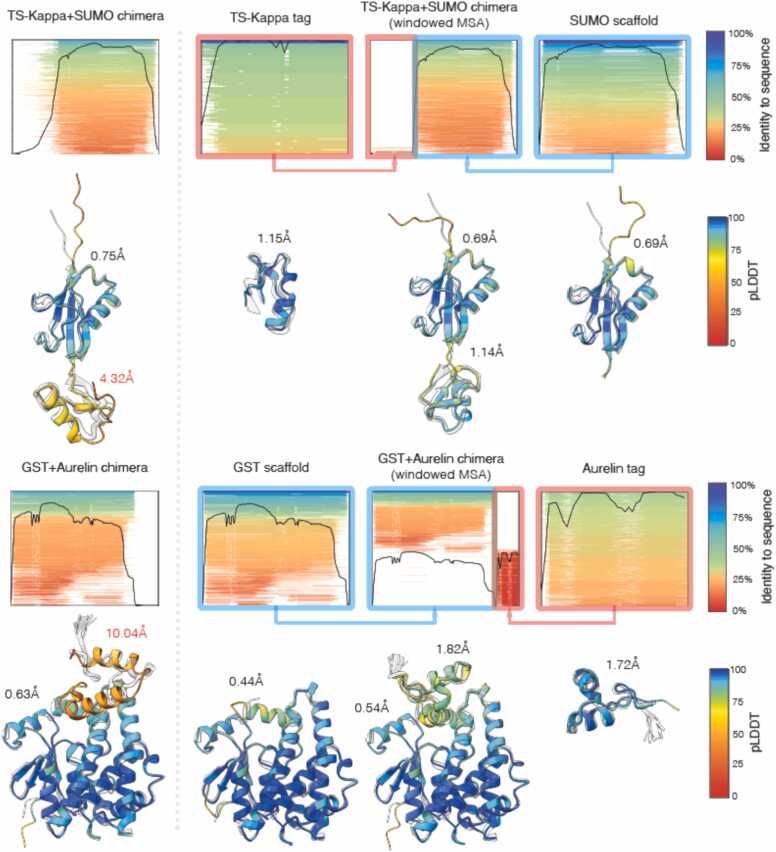
**AlphaFold-3 predicts exquisitely accurate structures for both the scaffolds and the tags individually, and fails to predict the tag region in the chimeric sequence when MSA coverage is lost.** Depicted on the left are the predicted structures for the peptide targets fused to the scaffolds. To the right and far right individual predictions of the peptide target and scaffold are shown. Between these individual predictions, the prediction for the peptide target fused to the scaffold when using a windowed MSA approach is shown. In this approach the MSA of the peptide target (framed in red) and the MSA of the scaffold (framed in blue) are combined into a chimeric MSA and used in prediction. Predicted structures are colored by the pLDDT confidence and superimposed on the experimentally determined structures (transparent white). RMSD of the tag prediction is reported in red. Below each predicted structure the MSA coverage is shown sorted and colored by identity to the query sequence. 1TSK+SUMO and GST+ 2LG4 chimeras are shown.

**Fig. 6:**
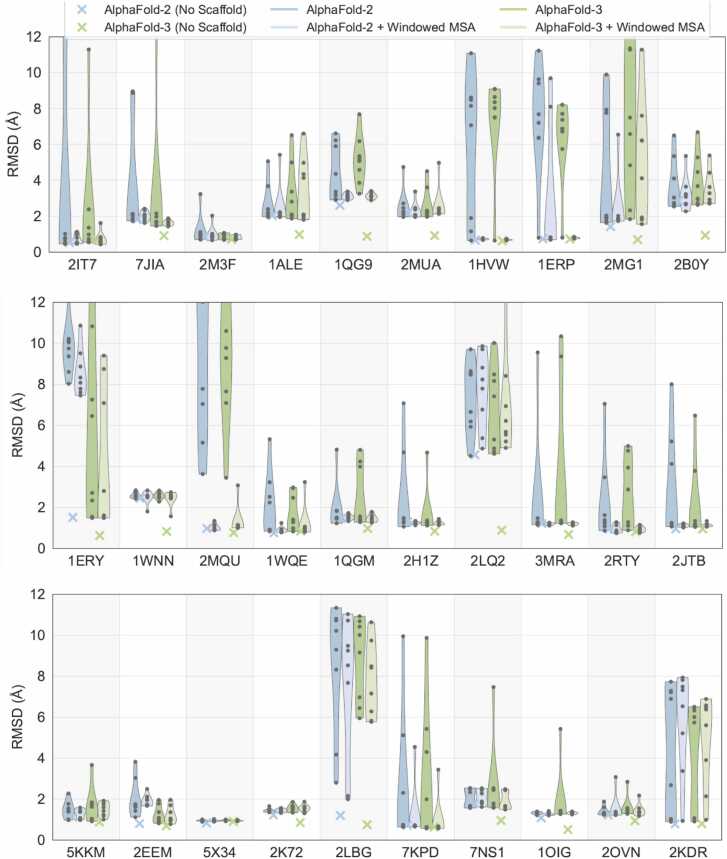
**Windowed MSA improves the prediction accuracy of AlphaFold-2 and AlphaFold-3.** The plot illustrates the RMSD distributions across multiple peptide tags for predictions made by AlphaFold-2 and AlphaFold-3, comparing results obtained with and without Windowed MSA. Each violin plot represents RMSD values for a particular target peptide (labelled by PDB ID) across all chimeric contexts tested (fusion to each the N- and C-terminus of the four different scaffold proteins). Predictions utilizing Windowed MSA consistently achieve comparable or improved accuracy compared to their non-windowed counterparts, often exhibiting significantly lower RMSD values and reduced variability. Baseline RMSD values of the individual peptide target prediction to the experimental structure are indicated by "x" markers.

We hypothesized that the inability of AlphaFold to accurately predict a peptide target when presented within a scaffold context – despite accurately predicting the tag in isolation – stems from inadequate representation of the peptide target MSA when presented as a fused sequence. To address this, we propose a method that combines the MSA obtained for individual regions; here combine the MSA from the individually queried scaffold with MSA from the individually queried peptide target. We ran AlphaFold-2 and AlphaFold-3 with windowed MSA predictions for all scaffold-target combinations and compare the accuracy of the updated predictions to those obtained using standard MSA. The significant improvement in scaffold context prediction accuracy can be appreciated in Fig. 5, that also visualises the MSA coverage and demonstrates that the windowed MSA approach improves the pLDDT confidence score output, and Sup. Fig. 3. The violin plot in Fig. 6 shows that improvement is common across all the tested peptide targets although cases where windowed MSA lead to a worsened prediction can be found (Sup. Fig. 4) and also cases where improved accuracy of peptide tag prediction is observed in one scaffold context but not another (Sup. Fig. 5).

## 4 Discussion

We compare the performance of three protein structure prediction algorithms, AlphaFold-2 Jumper et al. [6], AlphaFold-3 Abramson et al. [1], and ESMFold3 Hayes et al., [4] on in silico fusion sequences where each fusion partner is expected to fold independently and equivalently to the native form. Our main observation is that MSA dependent models such as AlphaFold-2 and Alphafold-3 commonly mispredict unnaturally fused sequences. This unnaturally fused sequence cannot be completely captured in a single MSA search and so prediction accuracy is lost for the region that loses coverage. We show that this limitation can be overcome by breaking down the MSA into windows and when the MSA for each of the fused components is provided to Alphafold, prediction accuracy is restored. We also show that whilst ESM approaches Rives et al. [14]; Rao et al. [13]; Hayes et al. (2024) do not generate explicit MSAs, they show the poorest prediction performance on these small and structured peptide targets, even in their isolated form.

It is likely that the degree of performance degradation in the fused context will depend on the lengths and relative lengths of the components being fused as well as their sequence depths, although this work did not address these parameters. However, this work demonstrates a clear benefit in segmenting the MSA into individually queried windows, when the window definitions were clear. Future work should explore how windows could be automatically defined for quick and effective prediction and this could find further usefulness in better predicting multidomain proteins. We also clarify that our work addresses only the task of accurately predicting the individual domain structures making the chimeric construct. A useful extension to this methodology would be implanting this step into the fuller pipeline that also addresses then the subsequent intradomain interactions however we defer this endeavor for future work.

## CRediT authorship contribution statement

**Bronstein Alex M:** Writing – review & editing, Writing – original draft, Visualization, Validation, Supervision, Resources, Project administration, Methodology, Investigation, Funding acquisition, Formal analysis, Data curation, Conceptualization. **Sanketh Vedula:** Writing – review & editing, Writing – original draft, Visualization, Validation, Project administration, Methodology, Investigation, Formal analysis, Data curation, Conceptualization. **Ailie Marx:** Writing – review & editing, Writing – original draft, Visualization, Validation, Supervision, Resources, Project administration, Methodology, Investigation, Funding acquisition, Formal analysis, Data curation, Conceptualization.

## Declaration of Competing Interest

The authors declare that they have no known competing financial interests or personal relationships that could have appeared to influence the work reported in this paper.
