# Supplementary material for: Improving prediction accuracy in chimeric proteins with windowed multiple sequence alignment
Source: Comput Struct Biotechnol J. 2025 Jul 23;27:3292–8. doi: 10.1016/j.csbj.2025.07.039 (PMC12328686; doi:10.1016/j.csbj.2025.07.039)
Supplement: Supplementary file 1 — Supplementary material [file mmc1.docx]

Supplementary Figures

Supplementary Figure 1 **Prediction accuracy is unaffected by linker length**. The structure of three different peptide tags fused to the N terminal of GFP, GST and SUMO were predicted with different GS repeat lengths linking between the tag and scaffold. Alignment of the tag region shows that the predicted structure is unaffected by the linker length.

Supplementary Figure 2. **Prediction accuracy is unaffected when tags are fused to both termini of the scaffold.** Chimeric sequences were created having peptide tags fused simultaneously to both the N and C termini of SUMO, GST and GFP. Each tag structure remains mis-predicted with Alphafold3 (red) and prediction accuracy is restored using the windowed MSA approach (green). The experimental structures of the individual scaffold and tag components are shown in gray.

Supplementary Figure 3 **Additional cases where the windowed MSA approach restores peptide tag prediction accuracy in the fused form**. Shown are cases where peptide tag structure is mis-predicted with Alphafold3 when fused to a scaffold protein (red) and prediction accuracy is restored using the windowed MSA approach (green). The experimental structures of the individual scaffold and tag components are shown in gray.

Supplementary Figure 4 **Cases where the windowed MSA approach worsens peptide tag prediction accuracy in the fused form**. Shown are cases where peptide tag structure is deteriorated using the windowed MSA approach (green) compared to the prediction with Alphafold3 (red). The experimental structures of the individual scaffold and tag components are shown in gray.

Supplementary Figure 5 **Cases where peptide tag prediction accuracy differs depending on the scaffold context**. Shown are three pairs of peptide tags, each bound to a different scaffold. Each pair shows a case where Alphafold3 mis-predicted peptide tag structure (red) is restored using the windowed MSA approach (green) and another case where the prediction accuracy is not restored by the windowed MSA approach. The experimental structures of the individual scaffold and tag components are shown in gray.
